# Change trend and gender differences in disability-free life expectancy among older adults in China, 2010–2020

**DOI:** 10.3389/fpubh.2023.1167490

**Published:** 2023-05-10

**Authors:** Min Lu, Xuehui Wang, Kaijun Shen, Chengpeng Ji, Wenxia Li

**Affiliations:** ^1^School of International Economics and Trade, Shanghai Lixin University of Accounting and Finance, Shanghai, China; ^2^School of Social Development and Public Policy, Fudan University, Shanghai, China; ^3^School of Public Administration, Hangzhou Normal University, Hangzhou, China; ^4^School of Business, Shanghai Jian Qiao University, Shanghai, China

**Keywords:** life expectancy, health life expectancy, disability-free life expectancy, gender differences, older adults, China

## Abstract

**Objective:**

The goal of the present study was to investigate gender differences in disability-free life expectancy (DFLE) and DFLE/LE ratio among older adults in China; portray changing trend from 2010 to 2020; and discuss the implications for public policies.

**Methods:**

Mortality data and disability rate data were derived from the Sixth China Population Census in 2010 and the Seventh China Population Census in 2020. The study assessed disability status of older adults based on self-assessment health in the above censuses. Life table and Sullivan method were used to estimate LE, DFLE, and DFLE/LE ratio by gender.

**Results:**

DFLE increased from 19.33 to 21.78 years for 60-year-old males, while from 21.94 to 24.80 years for 60-year-old females, from 2010 to 2020, respectively. DFLE/LE ratio was 96.40% for 60-year-old males and 94.86% for 60-year-old females in 2010, while DFLE/LE ratio was 96.63% for 60-year-old males and 95.44% for 60-year-old females in 2020, respectively. In terms of gender differences in DFLE/LE ratio, men aged 60 are 1.19 percentage points higher than women at the same age; men aged 70 are 1.71 percentage points higher than women; men aged 80 are 2.87 percentage points higher than women.

**Conclusion:**

From 2010 to 2020, the DFLE of China’s male and female older adults increased simultaneously with the increase of LE, and the DFLE/LE ratio also increased. However, the DFLE/LE ratio of female older adults is lower than that of male at the same age, and this gender difference is narrowing over the decade but has not yet been eliminated, especially the health disadvantage of female older adults among the oldest old age group (age 80 and above) is more prominent.

## 1. Introduction

Along with the unprecedented socio-economic development that has taken place globally over the past 50 years, people around the world are living longer (1). However it is uncertain whether an increase in life expectancy will result in an increase in healthy life expectancy (2). Meanwhile there is evidence that health life expectancy differs by gender. In general, women live longer than men, but are less physically and mentally healthy than men, and have a higher burden of morbidity or disability than men over a longer survival period (3, 4). Time spent in disability-free or disability (DFLE or DLE) plays a critical role in the use of public health services (5). This article explores the change trend and gender differences in disability-free life expectancy among older adults in China from 2010 to 2020, the results may serve as a guide for public policies in the country.

Since China entered the aging society in 2000, the aging process has been deepening, and the number and proportion of older adults has been gradually increasing (6). The data from the Seventh China Population Census show that in 2020, China’s population aged 60 and older has reached 260 million and the population aged 65 and older has reached 190 million, accounting for 18.7 and 13.5% of the country’s total population, respectively (7, 8), making it the country with the largest older adults population in the world. The aging process in China will continue to advance in the future, and it is expected that by 2050, there will be about 360 million people aged 65 and above in China, accounting for 26.1% of the total population (9). The large size and the increasing proportion of older adults will have a multi-dimensional effect and continuous pressure on all aspects of China’s economy and society, affecting the own retirement lives of older adults, increasing the burden on their families, and increasing the pressure on public service provision (6), however also bringing opportunities for the development of the “silver hair economy” and related new scientific and technological advances. The health status of older adults is an important factor in an aging society, and healthy aging is an important measure to implement the national strategy to actively cope with population aging.

Chinese population has experienced a health transformation and rising life expectancy, and the life expectancy has been increasing from 67.77 years in 1981 to 77.93 years in 2020 (10). The transformation has been attributed to China’s economic and social development, rising living standards, and improved healthcare conditions. However, the changes of life expectancy are unable to reflect the changes of health status of population (11, 12), so healthy life expectancy should be used. Healthy life expectancy (HLE) is an indicator that developed on the basis of life expectancy (LE). Life expectancy is composed of lengths of time spent in different states of health until death. These lengths of time spent in healthy states are healthy life expectancies. Life expectancy can reflect the length of life, while healthy life expectancy can reflect the quality of life (13). Longevity is a success for human society and brings with it the costs and benefits of success. On the one hand, advances in medications, lifestyle, and socioeconomics might improve ability in activities of daily living, that is, benefits of success. On the other hand, lifespan extension might expand disability in physical and cognitive functioning, as more frail individuals survive with health problems, that is, costs of success (14). This has led to a variety of health and social needs of older adults. Therefore, an accurate measure of the health status and HLE of older adults is of great significance for the older adults and their families to reasonably plan their life after old age, for the government and society to optimize the allocation of care resources and improve care policies of older adults.

Healthy life expectancy is an important indicator widely recognized to evaluate the health level of a population. Healthy life expectancy (HLE) is the number of remaining years at a particular age that an individual can expect to live in a healthy state (however health may be defined) unaffected by disease, death and dysfunction (6). The theoretical concept of HLE as health indicators was proposed by Sanders (15) and the first example was published in a report of the US department of Health Education and Welfare using a method devised by Sullivan (16). There are two main methods for estimating healthy life expectancy: the Sullivan method and multistate method. The multistate method requires longitudinal data to provide the transition rates between health states, while the Sullivan method uses more readily available data of current prevalence (13). In addition, based on the above two basic methods, many extension methods and computer programs have been proposed in recent years (17). Indeed, the Sullivan method has been used the most and generally can be recommended for its simplicity, relative accuracy and ease of interpretation (13, 18–21). Many empirical studies on healthy life expectancy usually use the functional ability to perform activities of daily living as an indicator of health status, and the HLE calculated on this basis is also known as Disability-Free Life Expectancy (DFLE) (13, 16). HLE is a more accurate indicator of the health status of a population than LE, and has been recommended by the World Health Organization to reflect the overall health status of the population.

Regarding the change trend in healthy life expectancy, there are three main theoretical hypotheses. The first one is the theory of “a compression of morbidity” proposed by Fries, which advocates that with socio-economic development, improved medical technology and better lifestyle, the population can live longer while delaying the onset of chronic diseases and disability, thus the time spent in morbidity or disability before death (i.e., DLE) is compressed (22). In contrast, Gruenberg proposed “an expansion of morbidity” hypothesis, which supports that the increase in life expectancy is accompanied by an increase in the survival of the frail, so the time spent in morbidity before death is expanding (23). In reality, these two trends might coexist and interplay, and the theory of “a dynamic equilibrium” has been proposed by Manton to help understand the association between morbidity and increasing life expectancy (24). In China, studies focusing on the HLE of the population have been gradually enriched since the 1990s, and these three theoretical hypotheses have been argued in related studies (25–27). Empirical studies on the above theoretical hypotheses in the country and abroad have not reached consistent conclusions so far.

Gender differences in health status and healthy life expectancy have been demonstrated in previous researches (3, 4, 28). Researches on gender differences in health in many countries have brought to light an important paradox: women report worse self-rated health than men, but women are less likely to die than same-aged men throughout life; women have longer LE than men, but their HLE/LE ratio is lower than that of men (29, 30). This is called the female–male health–survival paradox (31). There are several possible explanations for this paradox, and many explanations are rooted in biological, sociological, and psychological explanations. There may be multiple causes, including fundamental biological differences between the sexes, such as genetic factors, immune system responses, disease patterns, and more. From a socio-economic perspective, there are differences between women and men in many areas such as education, employment, income and accessibility of health services (32–34). Differences in lifestyle and behavior may also play a role, such as smoking, risk taking and unwilling to follow treatment (31, 35).

Previous studies have found that China’s female older adult is not as healthy as men, despite having a higher life expectancy. Older women have lower levels in activities of daily living (ADL) than men (36), and are more likely to follow a trajectory of disability (37). Particularly at advanced ages (80 years old and above), Chinese older women have a higher rate of disability during survival and are more likely to experience prolonged suffering before death than men (27). There are limited studies on HLE or DFLE of older adults in China, particularly those focusing on gender differences and changes over time based on census data. A study based on the 2005 sample survey and the 2010 census found that the proportion of DFLE in LE of males is higher than that of females, and the proportion of DFLE in LE is expanding from 2005 to 2010 (38). Another study using data from the 2010 census found that the proportion of disability life expectancy (DLE) in LE is significantly higher in women than in men, older women had longer periods in poor health than men (11). In addition, there are studies of healthy life expectancy based on longitudinal surveys, such as Chinese Longitudinal Healthy Longevity Study (CLHLS) and China Health and Retirement Longitudinal Study (CHARLS), however, the data are all prior to 2019 (27, 39). Therefore, this paper aims to use the latest data from the 7th National Population Census in 2020 to reflect the new changes in HLE of the Chinese older adults from 2010 to 2020 and the evolution of trends over the decade, and to explore the related public policy improvement issues from the perspective of gender differences in HLE.

## 2. Data and methods

### 2.1. Mortality data and disability-free rate data

The data required are the age-specific mortality information, and the age-specific prevalence of the population in healthy and unhealthy states. The data were derived from the Sixth (2010) and the Seventh (2020) China Population Census, which has been organized by the National Bureau of Statistics of the People’s Republic of China every 10 years, and encompassed all 31 provinces in mainland China.

Mortality data are derived from above two censuses. The population in each age group and the number of deaths in the age group are obtainable from the two censuses. Based on this data, the age-specific mortality rates of the whole population by gender in 2010 and 2020 are calculated, respectively. The mortality rate by single years of age will be used to calculate the complete life table and total life expectancy. Regarding the quality of the mortality data from the two censuses, relevant studies have shown that in terms of the stability of age-specific changes in mortality, the quality of mortality data is improving in China, with higher quality mortality in the 2010 and 2020 censuses (40, 41). Of course, population mortality data are only statistical values of mortality rather than actual mortality, and statistical values are bound to have random errors. However, because of the extremely large base of China’s population, the random error in mortality is extremely small and the magnitude of change is extremely small (42).

In the above two censuses, the health status of older adults aged 60 and older was statistically classified by age and sex, and the health status is based on self-assessment by older adults and families. Self-assessment health is a simple and integrated evaluation indicator that can not only reflect personal health status but also integrate the subjective and objective aspects of health status (43). The indicator of self-assessment health makes it more likely that people will be able to assess their health holistically, taking into account various social, physical, and emotional factors that affect their health (44). Admittedly, the indicator of self-assessment health has its drawbacks, which lie in its subjective nature, and it is not as relatively objective as a doctor’s assessment of health status. In the two censuses, the health status of older adults was divided into four categories: (a) “Healthy”; (b) “Basically healthy”; (c) “Not healthy, but disability-free”; (d) “Not healthy and disabled.” Due to the characteristics of the Sullivan method, the health status of the population needs to be distinguished into two categories in this paper: disability-free and disability. Therefore, the first three categories [including: (a) “Healthy”; (b) “Basically healthy”; (c) “Not healthy, but disability-free”] from the censuses are combined and defined as disability-free older adults, and the fourth category [i.e., (d) “Not healthy and disabled”] is defined as disability older adults in this paper. On this basis, the age-specific disability-free rate of older adults in China by gender in 2010 and 2020 are calculated, respectively. The health life expectancy that we shall calculate is disability-free life expectancy in this paper. Disability will be defined as requiring help with one or more activities of daily living (ADLs) (13).

### 2.2. Sullivan method

The complete life tables with full age range (0 to 95+ years) are complied firstly. Four complete life tables are produced in this paper, including two life tables for men and women in 2010, and two life tables for male and female in 2020. Then, based on the current life tables and the disability-free rate of the older adults, Sullivan method is used to estimate the disability-free life expectancy of the older adults. With the Sullivan method, we can decompose the life expectancy (LE) obtained from the original life tables into disability-free life expectancy (DFLE) and disability life expectancy (DLE). We can clearly know the time spent in disability-free or disability during the survival period before death. And the analysis of DFLE/LE ratio allows us to explore the relationship between longevity and health, and the quality of life. European Concerted Action on the Harmonization of Health Expectancy Calculations in Europe (EURO-REVES) published a practical guide for the calculation of healthy life expectancy by the Sullivan Method (13). This research uses the method recommended by the above guide to measure DFLE. The steps to estimate the disability-free life expectancy of older adults using Sullivan’s method are as follows:

(a) Based on the initial complete life tables with the full age range (0–95+ years) for 2010 and 2020, the number of surviving person-years lived with disability-free of older adults aged 60 and above is estimated by using the disability-free data of older adults. The formula is as follows:


$$L_{x}DF=L_{x}*\pi_{x}$$

In the above formula, *L_x_* refers to the number of surviving person-years in the corresponding age in the life table, *π_(x)_* is a newly added indicator item in the life table, which refers to the disability-free rate in the corresponding age of older adults.

(b) Estimate the total number of surviving person-years lived with disability-free, and the formula is as follows:


$$T_{x}DF=\sum_{x}^{w}L_{x}DF$$

(c) The age-specific disability-free life expectancy of older adults aged 60 and above is estimated by the following formula, in which *l_x_* represents the number of people alive at that age.


$$DFLE_{x}=T_{x}DF/l_{x}$$

(d) Calculate the proportion of disability-free life expectancy on life expectancy among older adults by age and gender, that is, *DFLE_x_*/*LE_x_* ratio, where *LE_x_* refers to the total life expectancy or remaining life years in the corresponding age.

## 3. Results

### 3.1. Disability-free rate by age and gender

The disability-free rate of older adults gradually decreases with age, and this trend is consistent among the male and female older adults. The disability-free rate of older adults shows significant gender differences. In the same year of 2010 or 2020, the disability-free rate of old men is generally higher than that of old women of the same age. In terms of different age groups, the gap of disability-free rate between male and female is not obvious among the age group of young old (age 60–79); however among the age group of oldest old (age 80 and above), the gap of disability-free rate between male and female is gradually becoming apparent, and the older the age, the greater the gap between male and female older adults.

**Figure 1:**
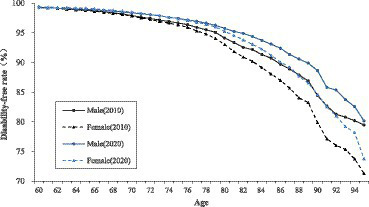
Disability-free rate of older adults by age and gender in China, 2010–2020.

From 2010 to 2020, the disability-free rate of both male and female older adults increased. The increase rate of old women is larger than that of old men, and compared with the young old women, the oldest old women have a more obvious increase in the disability-free rate, which also makes the gap between the male and female older adults narrow in 2020 compared with 2010. It is worth noting that in 2010, the disability-free rate of men is higher than that of women at the same age among the young old, but by 2020, the disability-free rate of the young old women will be reversed. However, the gender gap in the disability-free rate still exists objectively in 2020, especially in the age group of oldest old (age 80 and above), where older women are at a disadvantage in terms of disability-free rate significantly, compared to older men of the same age (Figure 1).

### 3.2. Trend in LE and DFLE by gender

In the same year, it can be seen that both the LE and DFLE gradually decrease with age, while DLE gradually increases with age. This trend is found in both male and female older adults. However, there are significant gender differences in the levels and changes in LE, DFLE and DLE of the older adults. The LE and DFLE of old women are generally longer than that of old men of the same age. Meanwhile, the DLE of old women is also longer than that of old men of the same age. Compared to gender differences in LE, gender differences in DFLE and DLE are relatively insignificant. In addition, among the age group of young old (age 60–79), the gender differences in LE, DFLE and DLE are greater and more pronounced; while among the age group of oldest old (age 80 and above), the gender differences in LE, DFLE and DLE are not as significant (Table 1).

**Table 1:** Disability-free life expectancy (DFLE) and DLE of older adults in China by age and gender, 2010–2020.

Age	Male			Female		
	LE	DFLE	DLE	LE	DFLE	DLE
In 2010						
60	20.05	19.33	0.72	23.13	21.94	1.19
65	16.24	15.52	0.73	18.92	17.73	1.19
70	12.78	12.05	0.73	15.02	13.84	1.19
75	9.86	9.12	0.74	11.63	10.44	1.19
80	7.43	6.67	0.76	8.72	7.51	1.21
85	5.71	4.93	0.79	6.57	5.35	1.22
90	4.55	3.70	0.85	4.95	3.72	1.23
95+	4.26	3.38	0.87	4.13	2.95	1.19
In 2020						
60	22.54	21.78	0.76	25.98	24.80	1.19
65	18.58	17.82	0.76	21.53	20.35	1.18
70	14.89	14.14	0.75	17.31	16.14	1.17
75	11.62	10.87	0.75	13.48	12.31	1.16
80	8.85	8.09	0.76	10.15	8.98	1.17
85	6.76	5.96	0.80	7.51	6.34	1.18
90	5.35	4.48	0.87	5.61	4.42	1.20
95+	4.83	3.87	0.96	4.69	3.46	1.23

The age in this table refers to the exact age, not the 5-year age group.

**Figure 2:**
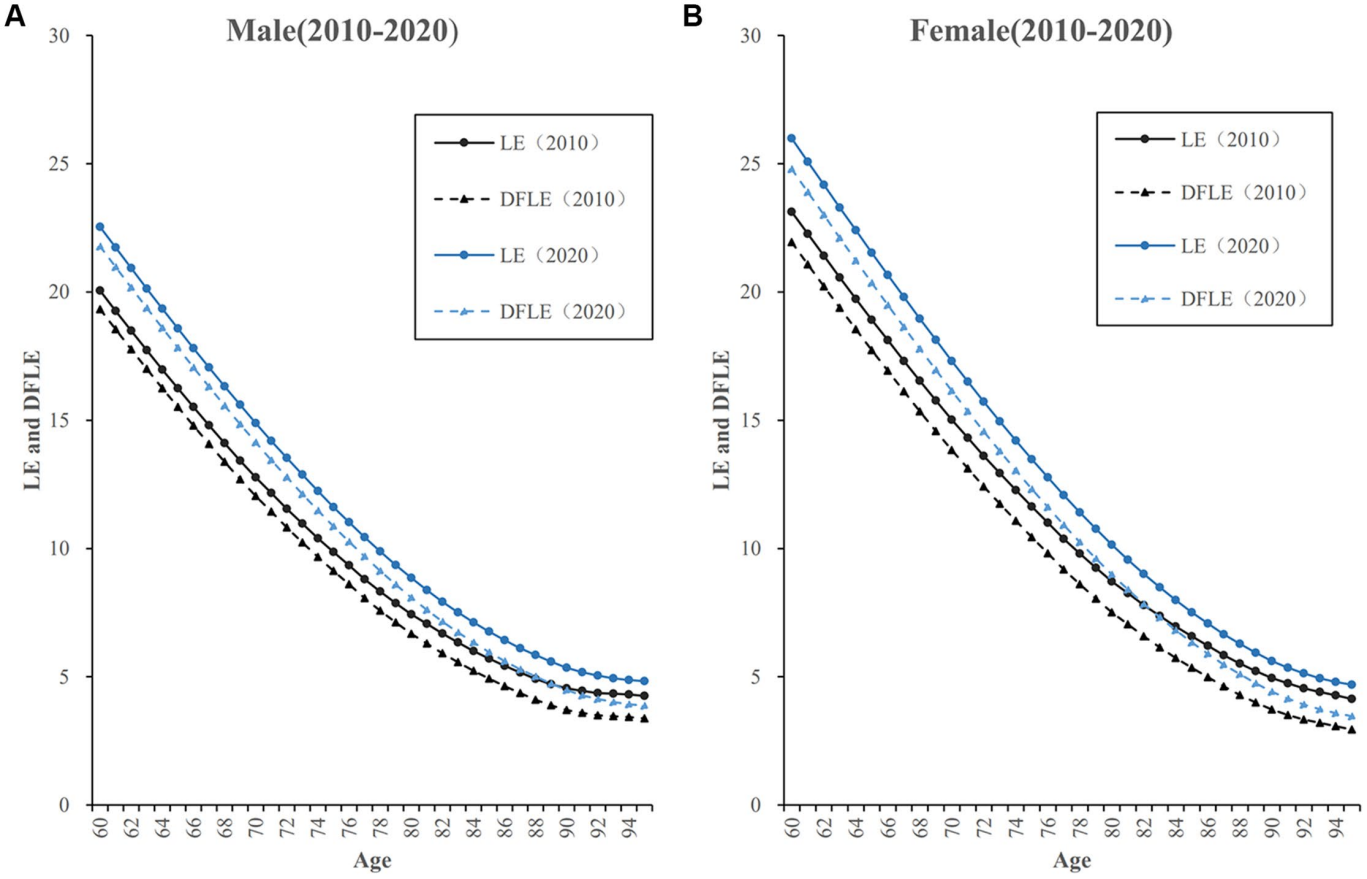
Life expectancy (LE) and DFLE of older adults by age and gender in China, 2010–2020. **(A)** LE and DFLE of male older adults in China, 2010-2020. **(B)** LE and DFLE of female older adults in China, 2010-2020.

Regarding the change trend over the decade, the LE for old men of the same age is increased from 2010 to 2020. Meanwhile, there are also increases in both DFLE and DLE for old men of the same age. For old women, the LE and DFLE of the same age are increased from 2010 to 2020, but the DLE of the same age slightly decreases in trend. In terms of the magnitude of the increase in LE and DFLE from 2010 to 2020, old women have a greater and more significant increase than old men; the age group of oldest old (age 80 and above) have a greater and more significant increase than the age group of young old (age 60–79). Compared to 2010, gender differences in LE and DFLE become larger in 2020, while the gender gap in DLE becomes smaller in 2020 (Table 1 and Figure 2).

### 3.3. Trend in the proportion of DFLE on LE by gender

The proportion of DFLE on LE (DFLE/LE ratio) gradually decreases with age, and the declining trend is more moderate for the age group of young old (age 60–79), while the declining trend is more distinct for the age group of oldest old (age 80 and above). This trend is present in both male and female older adults. Regarding the gender difference in the DFLE/LE ratio, it can be seen that the DFLE/LE ratio of old men is consistently higher than that of old women at the same age. Moreover, this gender gap in DFLE/LE ratio is not obvious among the age group of young old (age 60–79), while among the age group of oldest old (age 80 and above) this gender gap in DFLE/LE ratio is more obvious. There is a trend that the gender differences in DFLE/LE ratio become greater with increasing age (Table 2).

**Table 2:** Abbreviated life table of DFLE of older adults in China by gender, 2010–2020.

*x*	*l*_x_	*L*_x_	LE_x_	π_x_	L_x_DF	T_x_DF	DFLE_x_	DELE_x_/LE_x_ (%)
Male in 2010								
60	87,622	87,148	20.05	0.9934	86,570	1,693,638	19.33	96.40
65	82,005	81,287	16.24	0.9877	80,285	1,272,318	15.52	95.52
70	73,672	72,538	12.78	0.9789	71,010	887,466	12.05	94.28
75	61,125	59,620	9.86	0.9664	57,617	557,699	9.12	92.52
80	45,180	43,291	7.43	0.9411	40,742	301,410	6.67	89.79
85	27,204	25,553	5.71	0.9073	23,185	134,051	4.93	86.25
90	12,682	11,517	4.55	0.8443	9,724	46,940	3.70	81.41
95+	4,300	18,306	4.26	0.7946	14,547	14,547	3.38	79.46
Female in 2010								
60	93,309	93,026	23.13	0.9931	92,387	2,046,987	21.94	94.86
65	89,818	89,341	18.92	0.9875	88,224	1,592,641	17.73	93.72
70	84,096	83,270	15.02	0.9780	81,439	1,163,473	13.84	92.09
75	74,401	73,182	11.63	0.9630	70,472	776,665	10.44	89.74
80	60,367	58,512	8.72	0.9301	54,424	453,385	7.51	86.14
85	41,294	39,354	6.57	0.8805	34,652	221,060	5.35	81.43
90	22,683	20,978	4.95	0.7989	16,759	84,382	3.72	75.10
95+	8,911	36,834	4.13	0.7131	26,267	26,267	2.95	71.31
Male in 2020								
60	90,821	90,431	22.54	0.9923	89,730	1,978,358	21.78	96.63
65	86,321	85,773	18.58	0.9895	84,873	1,538,675	17.82	95.94
70	79,741	78,917	14.89	0.9834	77,610	1,127,635	14.14	94.95
75	69,894	68,662	11.62	0.9742	66,891	759,523	10.87	93.53
80	55,981	54,330	8.85	0.9574	52,017	452,887	8.09	91.38
85	38,315	36,460	6.76	0.9309	33,939	228,351	5.96	88.19
90	20,951	19,423	5.35	0.8862	17,214	93,845	4.48	83.67
95+	8,555	41,354	4.83	0.8016	33,150	33,150	3.87	80.16
Female in 2020								
60	95,791	95,612	25.98	0.9939	95,027	2,375,511	24.80	95.44
65	93,594	93,303	21.53	0.9915	92,508	1,904,999	20.35	94.53
70	89,851	89,333	17.31	0.9849	87,987	1,450,537	16.14	93.24
75	83,215	82,292	13.48	0.9735	80,114	1,024,724	12.31	91.37
80	72,033	70,551	10.15	0.9524	67,195	647,177	8.98	88.50
85	54,816	52,793	7.51	0.9121	48,151	347,363	6.34	84.34
90	33,816	31,671	5.61	0.8463	26,802	149,357	4.42	78.70
95+	15,033	70,557	4.69	0.7378	52,055	52,055	3.46	73.78

*x* represents age; *l*_x_ represents the number of people alive at that age; *L*_x_ represents the number of surviving person-years; LE_x_ represents life expectancy; π_x_ represents the disability-free rate at age x; L_x_DF represents the number of surviving person-years lived with disability-free; T_x_DF represents the total number of surviving person-years lived with disability-free; DFLE_x_ represents to disability-free life expectancy at age x; DELE_x_/LE_x_ (%) represents the proportion of disability-free life expectancy on life expectancy. This table is an abbreviated life table with excerpts only for age 60, 65, 70, 75, 80, 85, 90, and 95+, not a complete life table with the full age range (0 to 95+ years).

**Figure 3:**
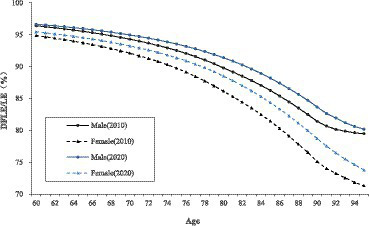
DFLE/LE (%) of older adults by age and gender in China, 2010–2020.

From 2010 to 2020, the DFLE/LE ratio for both male and female older adults have increased to varying degrees. In terms of the extent of improvement in DFLE/LE ratio, old women show a greater lift than old men. Meanwhile, the age group of oldest old (age 80 and above) shows a greater lift than the age group of young old (age 60–79). As can be seen, the gender gap in DFLE/LE ratio has narrowed over the decade from 2010 to 2020. However, in 2020 the gender gap in DFLE/LE ratio is still significant across all age groups, especially for the age group of oldest old (age 80 and above). Combining the data about LE and DFLE from the previous section, it can be seen that old women have longer LE and DFLE but have lower DFLE/LE ratio, while old men have shorter LE and DFLE but have higher DFLE/LE ratio. That is to say, although the female older adults survives longer than the male older adults, the quality of survival is worse than that of the male older adults (Figure 3).

## 4. Discussion

This paper has examined the LE and DFLE of male and female older adults in China from 2010 to 2020. Our results show that along with an increase in LE from 2010 to 2020, the DFLE of both genders has increased, and the DFLE/LE ratio of older adults at the same age also has increased to varying degrees. The result of our study supports the theoretical hypothesis of “a compression of morbidity” (22). It is consistent with the 1986–1995 situation in Japan (45) and the 2005–2011 situation in South Korea (46) and the 1980–1990 situation (among those of higher educational status) in the United States (3) and other countries and regions (47, 48). It can be assumed that the health status of China’s older adults has improved. Several contextual factors could explain these trends, including rapid economic and social growth, continued advancements in medical and healthcare services (14), and changes in population reproduction patterns and disease spectrum (6, 49). Improved health and longevity among older adults may lead to “health dividends” or “benefits of success” (14) for the individual, family, and society (50). For older adults, the benefits of improved health include better quality of life, increased societal participation, and more efficient utilization of human capital (49). For families, the benefits entail reduced caregiving burden as family care remains the primary form of support for older adults in China. For society and the state, improved health and longevity among older adults may reduce the impact of population aging on public policies and service systems (51). However, the observed improvements may also incur “costs of success,” as an increasing size of older adults survive longer with health problems (14). Therefore, changes in health and longevity are complex and multifaceted phenomena that require a comprehensive approach to address their implications.

Our study indicates that the variation in DFLE of older adults shows significant gender differences. From 2010 to 2020, the disability-free rate of older adults of both genders increased, and the disability-free rate of old men is higher than that of old women of the same age. Moreover, while the DFLE/LE ratio increased for both genders during the decade, the increase was greater for women, suggesting a narrowing of the gender gap. However the DFLE/LE ratio of old men is consistently higher than that of old women at the same age. The health disadvantage in DFLE/LE ratio of female older adults is still significant in 2020, particularly among the oldest old age group (age 80 and above). It is evident from our study that improvements in health and longevity in China have had a positive impact on the DFLE of both male and female older adults, albeit with gender-specific differences. While women generally live longer than men, our findings reveal that women’s longer lives do not necessarily translate to healthier lives. Our findings are consistent with prior researches conducted in China between 2005 and 2010 (27, 36–38), utilizing census data (11, 38) or sample survey data (27, 39). Additionally, gender differences in healthy life expectancy have been noted in other regions, such as the European Union, where females are expected to have longer periods with activity limitations than males (28). Similarly, during the 1986–2004 period in Japan, the proportion of expected years in good or average health (HLE) in the life expectancy of older women was lower than that of older men (42).

The gender differences observed in DFLE and DFLE/LE ratio suggest that the health needs of female older adults are more significant and long-term compared to their male counterparts (51). However current social welfare policies for older adults are predominantly gender neutral, but policies that ignore the fact that men and women have different socially determined roles, responsibilities, and capabilities are essentially gender-blind and can exacerbate existing gender inequalities (52). Therefore, it is essential to incorporate a gender perspective in the formulation of public policies and allocation of public services to eliminate health disparities (37). Moreover, from a perspective of life cycle, it is imperative to consider health problems faced by older adults as the result of a range of past experiences, including healthcare, housing conditions, hygiene practices, and education, and not merely limited to old age (5, 51). Thus, addressing older women’s health disparities requires a focus on promoting women’s rights and equality throughout their entire life cycle (52). To this end, there is a need to improve the disadvantageous position of women in terms of nutrition, education, and health services during early years, and reduce disparities in employment, income, and family caregiving in adulthood, to enhance women’s health and socio-economic accumulation across their life span.

## 5. Conclusion

Our study presents an analysis on disability-free life expectancy (DFLE) among Chinese older adults, with a particular emphasis on gender differences and change trends over the 2010–2020 period. It is found that along with an increase in LE, the DFLE and DFLE/LE ratio have increased for both genders over the decade. This result supports the theory of “a compression of morbidity.” Meanwhile, there are significant gender differences in DFLE among older adults in China. Female older adults have longer LE and DFLE but lower DFLE/LE ratio, while male older adults of the same age have shorter LE and DFLE but higher DFLE/LE ratio. It seems that for female older adults, the length of life is longer but the quality of life is worse and less healthy; for male older adults, the length of life is shorter but the quality of life is better and more healthy. Although the gender gap in DFLE/LE ratio has narrowed over the decade, gender differences still exist significantly in 2020, especially among the age group of oldest old (age 80 and above). These findings have important implications for policy development. Health promotion strategies should be adopted to sustainably improve the functional ability of daily living and overall health level for both female and male population in old age. Policy efforts are necessary to eliminate gender disparities in the quantity and quality of life years.

## Data availability statement

The data were derived from the Sixth (2010) and the Seventh (2020) China Population Census, which has been organized by the National Bureau of Statistics of the People’s Republic of China every 10 years, and encompassed all 31 provinces in mainland China.

## Author contributions

ML wrote the original manuscript. XW and KS made tables and figures. CJ and WL revised and advised the manuscript. All authors contributed to the article and approved the submitted version.

## Funding

This study was funded by major project of the National Social Science Foundation of China, grant number 20ZDA077 and youth project of the National Social Science Foundation of China, grant number 19CRK011.

## Conflict of interest

The authors declare that the research was conducted in the absence of any commercial or financial relationships that could be construed as a potential conflict of interest.

## Publisher’s note

All claims expressed in this article are solely those of the authors and do not necessarily represent those of their affiliated organizations, or those of the publisher, the editors and the reviewers. Any product that may be evaluated in this article, or claim that may be made by its manufacturer, is not guaranteed or endorsed by the publisher.
